# Between Deficiency and Excess: The Dual Role of Selected Dietary Supplements in Immune Health

**DOI:** 10.7759/cureus.104014

**Published:** 2026-02-21

**Authors:** Natalia Skrzypska, Wiktoria Glowacka-Kaminska, Justyna Wróblewska, Olga Wojtczak, Kacper Zagaja, Jakub Tarczykowski, Szymon Stupnicki, Maja Karminska, Krystian Czernikiewicz

**Affiliations:** 1 Hospital Medicine, University Clinical Hospital in Poznan, Poznan, POL; 2 Internal Medicine, Hipolit Cegielski Poznan (HCP) Medical Center, Poznan, POL; 3 General Practice, University Clinical Hospital in Poznan, Poznan, POL; 4 Outpatient, Uniwersytecki Szpital Kliniczny w Poznaniu, Poznan, POL; 5 General Practice, Wojewódzki Szpital Wielospecjalistyczny im. dr. Jana Jonstona w Lesznie, Leszno, POL; 6 Hospital Medicine, F. Raszeja Municipal Hospital, Poznan, POL

**Keywords:** dietary supplements, immune system, selenium, vitamin c, zinc

## Abstract

The immune system is a complex, tightly regulated network maintaining host defense, self-tolerance, and physiological homeostasis. Its effectiveness depends on the coordination of innate and adaptive responses, both significantly influenced by nutritional status. Recently, dietary supplements have gained attention as modulators of immune function, particularly in populations at risk of micronutrient deficiencies. This narrative review evaluates current evidence on the role of zinc, selenium, and vitamin C in immune health. These micronutrients were selected for their established roles in immune cell development, antioxidant defense, and inflammatory regulation. The review analyzes experimental, clinical, and epidemiological studies to assess their mechanisms, clinical efficacy, and safety. Zinc is essential for both innate and adaptive immunity, playing a pivotal role in T-lymphocyte maturation, macrophage and neutrophil function, and cytokine production. While zinc deficiency increases infection susceptibility, supplementation benefits primarily those with deficient or high-risk status. Selenium, a key component of selenoproteins like glutathione peroxidase, supports antioxidant defense and modulates cell-mediated immunity. Evidence indicates that selenium may enhance immune function in deficient individuals; however, excessive intake carries a narrow therapeutic window and potential toxicity. Vitamin C contributes to defense by maintaining epithelial barrier integrity, supporting leukocyte chemotaxis, and modulating oxidative stress. While routine high-dose supplementation offers limited benefits in healthy individuals, adequate intake remains crucial for those with low dietary consumption.

This review highlights the risks associated with indiscriminate supplementation. While zinc, selenium, and vitamin C are indispensable for optimal immunity, their use should be individualized and targeted toward correcting confirmed deficiencies. A personalized, evidence-based approach remains essential for supporting immune health while minimizing potential risks.

## Introduction and background

The human immune system is a sophisticated biological network designed to maintain physiological homeostasis by distinguishing between self-antigens and foreign pathogens. Its primary objective is to execute rapid and effective defense mechanisms against infectious agents while preventing auto-inflammatory damage to the host’s own tissues [1]. This defensive architecture is traditionally categorized into two synergistic arms: innate and adaptive immunity.

The innate immune system functions as the first line of defense, utilizing a conserved set of pattern recognition receptors to identify microbial structures, thereby triggering immediate inflammatory responses and priming the subsequent adaptive response [2]. In contrast, adaptive immunity is characterized by its high specificity and the generation of immunological memory. Orchestrated primarily by B and T lymphocytes, this system ensures an enhanced and accelerated response upon subsequent exposures to previously encountered antigens [3].

Maintaining the delicate equilibrium between immune activation and self-tolerance is a highly regulated process. This balance is governed by a multitude of complex factors, including cytokine signaling, regulatory T-cell activity, and, increasingly recognized, the metabolic and nutritional status of the individual [4].

In recent years, the role of micronutrients in modulating immune resilience has gained significant clinical interest. While a balanced diet remains the cornerstone of health, certain physiological conditions, age-related changes, or geographical factors can lead to nutritional inadequacies that necessitate intervention.

Dietary supplements, a diverse category of products ranging from single vitamins and minerals to complex botanical extracts, are increasingly utilized to address these deficiencies or to enhance specific biological functions [5]. Although they offer potential benefits in managing oxidative stress, improving cognitive function, and modulating immune performance, their efficacy is often debated in the absence of clinical deficiency [6].

Current literature emphasizes that while supplements can support the immune system, they must not be perceived as a replacement for dietary diversity [7]. Among the myriad of available supplements, zinc, selenium, and vitamin C have emerged as three of the most critical elements involved in immune maintenance. This review aims to synthesize current evidence regarding the clinical impact of these specific micronutrients on immune function, while critically evaluating the risks associated with their over-supplementation and the lack of stringent industry regulation.

## Review

Methods

Study Design

This narrative review summarizes current evidence on the role of selected dietary supplements, zinc, selenium, and vitamin C, in immune system function. A narrative approach was chosen to integrate mechanistic, clinical, and epidemiological data. A targeted literature search was conducted across the PubMed (n=145) and Google Scholar (n=65) databases, including articles published up to early 2025. Search terms included combinations of “immune system,” “immunity,” “zinc,” “selenium,” “vitamin C,” and “dietary supplements.” After removing duplicates and screening 160 unique records by title and abstract, 60 full-text articles were assessed for eligibility. Additional relevant studies were identified through manual screening of reference lists. The final selection of 34 references included experimental studies, randomized controlled trials, observational studies, and systematic reviews. Priority was given to human studies, with selected in vitro and animal studies included to support mechanistic explanations. Findings were synthesized qualitatively, with an emphasis on baseline nutritional status, clinical efficacy, and the risks associated with supplementation.

Risk of Bias

As a narrative review, this study is subject to inherent limitations, including potential selection bias, as study inclusion was based on relevance rather than a predefined systematic protocol. Publication bias may also be present, given the greater likelihood of publishing studies with positive findings. Heterogeneity among included studies, regarding study design, populations, baseline micronutrient status, supplementation doses, and outcome measures, limits direct comparability and causal interpretation. Additionally, confounding factors were not uniformly controlled across observational studies. To address these limitations, this review prioritizes evidence from randomized controlled trials and meta-analyses when available and emphasizes cautious interpretation of findings, particularly in individuals without confirmed micronutrient deficiencies.

Definition and role of the immune system

The immune system is a structure responsible for quickly recognizing and effectively protecting the human organism against foreign pathogens while avoiding attacks on the body’s own cells [1]. The human immune defense possesses two primary defensive lines that protect against pathogenic infectious agents: innate immunity and adaptive immunity. The innate immune system represents a key protective barrier by utilizing a restricted set of receptors that recognize conserved microbial structures. This activation induces a rapid inflammatory response and facilitates the engagement of adaptive immunity [2]. The adaptive immune system comprises a network of cells, molecules, and effector mechanisms that utilize specialized receptors to detect and respond to specific antigens. Those antigens can be derived from entities outside the body, such as pathogens and allergens, or from internal sources, such as tumors and self-tissues. A defining feature of adaptive immunity is the development of immune memory, the ability of lymphocytes to rapidly and accurately react to antigens previously encountered, providing enhanced or complete protection against subsequent exposures [3]. The primary cellular components of adaptive immunity are lymphocytes, specifically T cells and B cells. Conventional αβ T cell populations are subdivided into CD4+ helper T cells and CD8+ cytotoxic T cells. CD4+ T cells carry out a variety of effector functions through both soluble mediators and direct cell-to-cell interactions, whereas CD8+ T cells primarily mediate their effects by directly killing target cells. B cells secrete soluble effector molecules called antibodies and also serve as antigen-presenting cells (APCs), which present specific antigens to T cells [3]. The immune system must maintain a careful balance between tolerance to self-antigens and defense against infections. Precise molecular mechanisms that control immune activation and prevent excessive or inappropriate immune responses are responsible for achieving this balance. These mechanisms are complex and include several factors such as immunometabolism, cytokine signaling, immune checkpoints, regulatory T cells, and epigenetic changes [4].

Definition and impact of dietary supplements on health

The term “dietary supplement” refers to a broad and diverse group of products that are vital for health but are either missing or insufficient in the diet, necessitating separate intake [5]. Dietary supplements cover a wide range of products, including multivitamins, single vitamins or minerals, proteins, amino acids, prohormones, herbal substances, joint support supplements, combination products, and other plant-, animal-, or synthetic-derived substances. They may be administered orally as powders, liquids, capsules, or tablets [6]. The potential health benefits of dietary supplements may include improving cognitive function, maintaining joint functioning, reducing the risk of cancer, modulating the immune system, reducing aging, protecting from cardiovascular diseases, maintaining bone mineral density, and improving reproductive health [5]. It is worth emphasizing that dietary supplements may be used to meet dietary needs or supplement nutritional deficiencies, but they are not intended to substitute for a healthy diet [7]. The aim of this research is to review current data on the role of selected dietary supplements, zinc, vitamin C, and selenium in the functioning of the immune system.

Zinc

Zinc is an essential trace element necessary for the optimal functioning of the human immune system. Deficiency in zinc impairs both innate and adaptive immunity, resulting in increased susceptibility to bacterial and viral infections and a more severe progression of infectious diseases [8]. Zinc is integral to the regeneration of the thymus and the subsequent maturation and function of T lymphocytes, the activity of macrophages and neutrophils, cytokine production, and the regulation of inflammatory responses and oxidative stress [8,9].

Individuals at increased risk of zinc deficiency, including older adults, malnourished individuals, patients with chronic inflammatory diseases, and those with HIV infection, may derive particular benefit from supplementation. Moderate zinc deficiency has been shown to rapidly impair both humoral and cell-mediated immunity, increasing susceptibility to opportunistic and common infections. Studies in these populations indicate that zinc supplementation is associated with significant reductions in inflammatory markers, such as C-reactive protein (CRP), interleukin-6 (IL-6), and tumor necrosis factor alpha (TNF-α), as well as increases in CD4+ lymphocyte counts [10].

Beyond its role in chronic inflammatory states, the clinical utility of zinc has been extensively scrutinized in the management of acute respiratory infections. The therapeutic rationale is based on zinc’s ability to inhibit viral replication and prevent the binding of pathogens to the nasal mucosa. A comprehensive systematic review and meta-analysis by Wang et al. (2020), which evaluated evidence from several randomized controlled trials, provided more definitive insights into this application. The authors demonstrated that zinc supplementation initiated within 24 hours of symptom onset could reduce the duration of common cold symptoms by an average of 2.25 days. However, the study also clarified important limitations: zinc intake did not significantly reduce the overall severity of symptoms, nor did it show efficacy as a prophylactic measure to prevent the onset of colds in healthy individuals. Furthermore, the review highlighted that the effectiveness is highly dependent on the formulation and dosage, with ionic zinc (e.g., zinc gluconate or acetate) appearing more effective than other forms [11].

Current recommendations emphasize achieving adequate dietary zinc intake (11 mg/day for adult men, 8 mg/day for adult women; upper intake level 25-40 mg/day depending on the authority). Therefore, dietary improvement, through increased intake of zinc-rich foods such as meat, seafood, and legumes, should be regarded as the first-line approach when feasible. Supplementation should be considered primarily in cases where zinc deficiency is documented or highly probable [12,13].

Further clinical trials are needed to clarify optimal dosing, formulation, and long-term safety, especially in diverse patient populations. Current recommendations favor the targeted correction of zinc deficiency over routine, universal supplementation [14,15].

Selenium and its significance in the immune response

Selenium exerts its immunomodulatory effects primarily through its incorporation into selenoproteins such as glutathione peroxidase and thioredoxin reductase. These enzymes protect immune cells from oxidative stress while regulating calcium flux and protein palmitoylation necessary for T-cell activation [16].

While the necessity of selenium is well-established, defining the optimal dosage for immunological enhancement remains a significant challenge. Research has yet to establish a definitive, universally applicable intake level that maximizes immune resilience without risking toxicity. This lack of a standardized dosing framework underscores the complexity of selenium’s bioactivity and the critical need for individualized assessment of nutritional status [16].

The strongest evidence for benefit comes from supplementing people who are already deficient in selenium. For those with normal selenium levels, taking high doses regularly does not show consistent or clear benefits.

In selenium-deficient individuals, supplementation may enhance immune function by increasing T lymphocyte proliferation and natural killer (NK) cell activity and shifting the Th1/Th2 balance toward a Th1-dominant response, which supports antiviral and antitumor immunity [17]. Populations at increased risk of selenium deficiency include individuals living in regions with low selenium content in soil (including parts of Europe, China, and Africa); pregnant women; older adults; patients with chronic diseases such as malabsorption syndromes or chronic kidney disease; individuals following restrictive diets (e.g., vegans or those on elimination diets); patients with COVID-19; and individuals recovering from severe illness [16,18].

Further insights into the relationship between selenium and immunity are provided by Fairweather-Tait et al. (2023), who conducted a systematic review focusing on high-level evidence from randomized controlled trials. Their analysis indicates that while selenium is integral to the structure of 25 selenoproteins, its specific impact on human immune parameters remains complex and often inconsistent. The authors found that the effects of selenium supplementation on humoral immunity appear negligible. Crucially, Fairweather-Tait and colleagues emphasize that these outcomes are highly dependent on the dosage and chemical form of selenium administered. They conclude that current experimental data are still too limited to establish a definitive dose-response relationship, underscoring the need for more targeted investigations into how baseline selenium status dictates the immune system's response to supplementation [19].

Crucial evidence regarding the safety of long-term supplementation is provided by the Nutritional Prevention of Cancer (NPC) randomized controlled trial, as reported by Stranges et al. (2007). In this long-term clinical study, the authors demonstrated that selenium supplementation not only fails to provide metabolic benefits in individuals with adequate baseline levels but may paradoxically be associated with an increased risk of developing type 2 diabetes. These primary findings highlight a narrow therapeutic window and suggest that in populations with high baseline serum selenium status, additional intake can disrupt glucose homeostasis. Consequently, this experimental evidence challenges the rationale for routine, universal supplementation in well-nourished populations [20].

In conclusion, selenium is crucial for an effective immune response, particularly in the context of cell-mediated immunity, and its deficiency is associated with impaired immune function. Selenium supplementation should therefore be considered only in individuals with confirmed deficiency or a clearly increased risk of inadequate selenium status.

Impact of vitamin C on immune system performance

Vitamin C, also known as ascorbic acid, is a common ingredient in supplements that are suggested to support the immune system, for example, during the common cold. What does this vitamin do that makes it so important for defending our health?

Beginning with the outermost part of the immune system, ascorbic acid is responsible for the functioning of the epithelial barrier, which is our first line of defense [21]. Vitamin C, through stimulation of ceramide synthesis, acceleration of keratinocyte differentiation, influence on the expression of barrier-related genes, and many other mechanisms, strengthens and tightens the skin barrier. An intact and healthy epithelial barrier hinders the entry of microorganisms into the body [22].

If a pathogen has already penetrated, ascorbic acid continues to perform its function. The publication by Bozonet and Carr explains how vitamin C affects leukocytes, which play a crucial role in the immune system, and their ability to detect and migrate towards pathogens [23]. It has been proven that neutrophils of patients with hypovitaminosis C manifest impaired migration toward the site of inflammation [24]. This results in poorer detection of microorganisms and less effective elimination of them.

Furthermore, vitamin C enhances phagocytosis performed by neutrophils, a process that involves the engulfment of pathogens. Research suggests that it is responsible for stimulating the oxidative burst, which produces reactive oxygen species to neutralize microbes [25].

Although only some of the functions of vitamin C have been discussed, its wide range of effects highlights the importance of proper vitamin C intake, which will be addressed in the following section. A daily intake of 100-200 mg of vitamin C allows healthy individuals to reach adequate plasma concentrations, which should be sufficient to meet the body’s general requirements. Since vitamin C is a water-soluble compound stored only in small amounts, regular and adequate consumption is essential to prevent hypovitaminosis C [26]. Surprisingly, epidemiological data indicate that low plasma vitamin C levels (< 23 μmol/L) are relatively common in Western populations. This suggests that, in certain circumstances, dietary intake is insufficient to provide an adequate dose of vitamin C [27].

While clinical toxicity from excessive vitamin C intake is less frequently documented compared to other micronutrients, significant risks regarding long-term high-dose supplementation have been identified. Evidence from a large-scale study by Ferraro et al. (2016) demonstrates a clear association between supplemental vitamin C intake and the development of nephrolithiasis (kidney stones). The authors found that men consuming 1000 mg or more of supplemental ascorbic acid per day had a significantly higher risk of incident kidney stones. This is attributed to the metabolic conversion of vitamin C into oxalate, which, when excreted in high concentrations, promotes the formation of calcium oxalate crystals in the urinary tract [28].

Consequently, these findings provide evidence that vitamin C supplementation must be a deliberate and carefully considered clinical decision rather than an indiscriminate daily habit.

Risk and limitations of dietary supplements

Although supplementation can offer moderate benefits in specific situations, it is also associated with potential downsides. Broome emphasizes that dietary supplementation, specifically selenium, yields significant benefits primarily, if not exclusively, in individuals presenting with pre-existing nutritional deficiencies [29]. In the absence of nutritional deficiencies, supplementation beyond the levels required for tissue saturation may fail to provide any additional immunological benefits. The research demonstrates that not only may such supplementation lack clinical benefit, but in certain instances, it may even lead to adverse effects or harm.

Excessive intake of specific micronutrients can disrupt the delicate homeostatic balance of the immune system. For instance, prolonged high-dose zinc supplementation is known to interfere with copper absorption, potentially leading to copper deficiency, which paradoxically impairs immune function and causes neutropenia [30]. Potential adverse effects, such as nausea and a metallic taste, should also be considered when zinc is used therapeutically [14].

Similarly, while selenium is vital for antioxidant defense, its therapeutic window is remarkably narrow; excessive levels can induce pro-oxidant effects, leading to selenium toxicity [31].

Another critical limitation is the "U-shaped dose-response curve" characteristic of many nutrients. Both deficiency and supra-physiological intake can result in immune dysfunction. Additionally, the reliance on supplements may create a "false sense of security," leading individuals to neglect fundamental health practices, such as a balanced diet, adequate sleep, and stress management, which are irreplaceable for a robust immune response [32].

Lastly, the lack of stringent regulation in the supplement industry often results in products with inaccurate labeling or undisclosed contaminants, posing further risks to the consumer. The regulatory framework for dietary supplements significantly differs from that of pharmaceuticals, as supplements are often classified as food products rather than medicines. Consequently, they are not subject to the same rigorous pre-market testing for safety and efficacy. Research has consistently identified discrepancies between labeled dosages and actual chemical composition, with some products containing sub-therapeutic levels or, conversely, potentially toxic concentrations of active ingredients [33]. Furthermore, independent laboratory analyses have frequently detected undisclosed contaminants, including heavy metals like lead and cadmium, or undeclared pharmacological agents. These quality control failures pose substantial risks, ranging from acute toxicity to chronic organ damage, and underscore the importance of choosing products certified by independent third-party organizations [34].

To provide a clear overview of the clinical implications discussed, Table 1 synthesizes the immunological roles, target populations, and safety profiles of the analyzed micronutrients.

**Table 1:** Summary of immunomodulatory micronutrients (functions, deficiencies, and supplementation risks).

Micronutrient	Primary Immune Function	Groups at High Risk of Deficiency	Risks of Excessive Intake	References
Zinc	T-cell maturation, macrophage/neutrophil activity, cytokine regulation, and inhibition of viral replication.	Older adults, malnourished individuals, chronic inflammatory disease patients, and HIV+ individuals.	Copper deficiency, neutropenia, impaired immune response, and metallic taste.	[8,-11,14]
Selenium	Antioxidant protection (selenoproteins), Th1/Th2 balance modulation, and T-lymphocyte proliferation.	Regions with low-soil selenium, pregnant women, restrictive diets (vegans), and chronic kidney disease patients.	Selenium toxicity, pro-oxidant effects, and increased risk of type 2 diabetes.	[16-20]
Vitamin C	Epithelial barrier integrity, neutrophil chemotaxis (migration), phagocytosis, and oxidative burst stimulation.	Smokers, the elderly, and individuals with low fruit/vegetable intake.	Potential kidney stone formation in predisposed individuals.	[21-27]

Future directions

As the understanding of immunometabolism evolves, future research must transition from broad population-based recommendations to more nuanced, individualized strategies. One of the most promising avenues is the integration of nutrigenomics, which examines how genetic variations influence an individual’s response to micronutrient intake. Identifying specific polymorphisms in genes responsible for zinc transport or selenoprotein synthesis could explain the heterogeneous responses observed in clinical trials and allow for truly personalized supplementation protocols.

Furthermore, the role of the gut-immune axis remains a critical area for investigation. Emerging evidence suggests that zinc and selenium significantly modulate the gut microbiota, which in turn trains systemic immune responses. Future studies should employ metagenomic sequencing to determine how micronutrient supplementation alters microbial diversity and intestinal barrier integrity, potentially offering new therapeutic targets for systemic inflammatory conditions.

Finally, there is an urgent need for the development and validation of functional biomarkers of nutrient status. Current diagnostic tools often measure static serum levels, which may not accurately reflect the intracellular availability of micronutrients during acute illness. Establishing "real-time" biomarkers of immune-specific nutrient saturation would enable clinicians to titrate dosages more precisely, maximizing host defense while strictly avoiding the thresholds of iatrogenic toxicity.

## Conclusions

The evidence confirms that while zinc, selenium, and vitamin C are indispensable for immune homeostasis, their clinical utility is strictly delimited by an individual’s baseline nutritional status, yielding significant benefits primarily in deficient populations. For instance, while zinc can reduce common cold duration by 2.25 days, it does not significantly lower infection incidence in nutrient-replete individuals, establishing that its biological utility is finite. Furthermore, the data reinforce a "U-shaped dose-response" paradigm, where supra-physiological intake poses iatrogenic risks such as zinc-induced neutropenia, selenium-related metabolic disturbances, or nephrolithiasis from excessive vitamin C. These physiological risks are exacerbated by the lack of stringent pharmacological regulation in the supplement industry, necessitating a cautious, evidence-based approach to product selection. In summary, the transition toward a personalized nutritional framework is essential, prioritizing targeted interventions over universal, high-dose recommendations. Future clinical perspectives should focus on integrating precise biomarkers, nutrigenomic data, and the gut-immune axis to develop safer, individualized protocols that maximize host defense while avoiding chronic toxicity.
